# Exploring the needs of children and caregivers to inform design of an artificial intelligence-enhanced social robot in the pediatric emergency department

**DOI:** 10.1017/cts.2023.608

**Published:** 2023-08-24

**Authors:** Fareha Nishat, Summer Hudson, Prabdeep Panesar, Samina Ali, Sasha Litwin, Frauke Zeller, Patricia Candelaria, Mary Ellen Foster, Jennifer Stinson

**Affiliations:** 1 Child Health Evaluative Sciences, The Hospital for Sick Children, Toronto, ON, Canada; 2 Department of Pediatrics, Faculty of Medicine & Dentistry, University of Alberta, Edmonton, AB, Canada; 3 Division of Emergency Medicine, The Hospital for Sick Children, Toronto, ON, Canada; 4 School of Computing, Engineering, and The Built Environment, Edinburgh Napier University, Edinburgh, SC, UK; 5 School of Computing Science, University of Glasgow, Glasgow, SC, UK; 6 Lawrence S. Bloomberg Faculty of Nursing, University of Toronto, Toronto, ON, Canada

**Keywords:** Artificial intelligence, social robotics, needs assessment, procedural distress, children, co-design

## Abstract

**Background & Objective:**

Socially assistive robots (SARs) are a promising tool to manage children’s pain and distress related to medical procedures, but current options lack autonomous adaptability. The aim of this study was to understand children’s and caregivers' perceptions surrounding the use of an artificial intelligence (AI)-enhanced SAR to provide personalized procedural support to children during intravenous insertion (IVI) to inform the design of such a system following a user-centric approach.

**Methods:**

This study presents a descriptive qualitative needs assessment of children and caregivers. Data were collected via semi-structured individual interviews and focus groups. Participants were recruited from two Canadian pediatric emergency departments (EDs) between April 2021 and January 2022.

**Results:**

Eleven caregivers and 19 children completed 27 individual interviews and one focus group. Three main themes were identified: A. Experience in the clinical setting, B. Acceptance of and concerns surrounding SARs, and C. Features that support child engagement with SARs. Most participants expressed comfort with robot technology, however, concerns were raised about sharing personal information, photographing/videotaping, and the possibility of technical failure. Suggestions for feature enhancements included increasing movement to engage a child’s attention and tailoring language to developmental age. To enhance the overall ED experience, participants also identified a role for the SAR in the waiting room.

**Conclusion:**

Artificial intelligence-enhanced SARs were perceived by children and caregivers as a promising tool for distraction during IVIs and to enhance the overall ED experience. Insights collected will be used to inform the design of an AI-enhanced SAR.

## Introduction

Children frequently experience pain and distress in the context of needle-related medical procedures, including vaccine administration and intravenous insertion (IVI). Without intervention, these experiences can lead to both short-term (e.g., procedure failure, delays in care) and long-term (e.g., healthcare avoidance, needle phobia) adverse consequences [1–3]. Both digital and non-digital tools (e.g., bubble blowing, virtual reality) have been shown to reduce children’s procedure-related pain and distress [4–6]. Socially assistive robotics (SARs) have the potential to be uniquely beneficial as a novel technology with potential to create a more immersive experience for children than other known digital distraction modalities. Several recent studies have shown promising results regarding the application of SAR to mitigate children’s procedure-related pain and distress [7,8].

**Figure 1.**
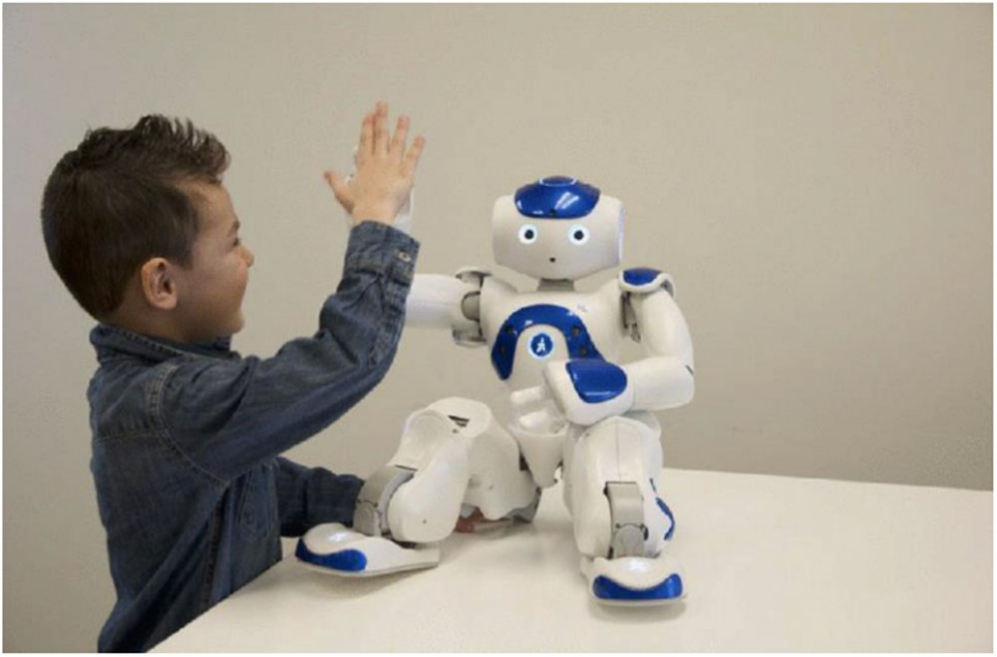
Child interacting with the Nao humanoid robot.

SAR encompasses robots designed to assist humans via interactive communication (Fig. 1) [9]. SARs have demonstrated benefits across various conditions, including stroke rehabilitation [10–14]. Within pediatrics, SARs have been increasingly implemented to ameliorate pain and distress, with positive outcomes [7,10–13]. Specifically, robots have been shown to reduce distress for needle-related procedures in the pediatric emergency department (ED) [7]. Similar results exist for pre-procedure and vaccination-related pain and distress [14,15].

The overall project objective is to develop an AI-enhanced SAR to enable autonomous action selection for a robot’s behaviors, thereby offering personalized, adaptive procedural support to children during IVI. To achieve this, the humanoid Nao robot (SoftBank Robotics) will serve as the physical form (22.5 inches tall, 12 pounds). This platform has been widely used in child-robot interaction studies [7,12,13,16]. Details of the overall project methodology have been previously published [17]. This current study specifically aimed to characterize children’s and caregivers’ (a) experiences of IVI-related pain and distress and (b) their perspectives surrounding integration of robots and AI within the pediatric healthcare context, to inform the design of this system.

While prior studies demonstrate clear potential for a SAR to reduce procedure-related pain and distress in children, existing systems are all significantly limited by their preprogramed nature – utilizing entirely scripted behavior or requiring real-time human input for behavior selection. This restricts their responsiveness and flexibility in the unpredictable pediatric clinical setting [8]. Artificial intelligence (AI), the ability of computer systems to make autonomous decisions and independently select appropriate actions, has potential to address this limitation. This study will explore the desired functionality of AI from the perspective of children and caregivers through user-centered co-design. This involves engagement of designers with stakeholders to conceptualize a system [18,19]. Use of co-design practices increases the likelihood of successful product implementation. This collaborative method has been frequently used in the design of AI-based systems and SAR for real-world applications, including within the pediatric domain [20–23].

## Methods

### Study design

We conducted a descriptive co-design study involving target end-users [24]. Participants completed either semi-structured individual interviews or focus groups. Institutional ethics approval was obtained from the Hospital for Sick Children’s [1000072883] and the University of Alberta’s Research Ethics Boards [Pro00097697].

Integration of novel technology into the clinical context requires careful consideration of related ethical issues and a moral commitment to patient well-being. The use of AI methodologies for SARs requires specific ethical consideration, given the associated unique challenges. To address this, we employed a vertically integrated ethics perspective, meaning that ethical inquiry was undertaken at the outset and prioritized throughout all phases of the study. Practically, this included incorporation of co-design questions related to the notions of ethics in SAR design. For example, we inquired about trustworthiness and other perceptions of the robot. Additionally, we recognize that notions of trust and privacy may differ among stakeholders – children, caregivers, and healthcare providers (HCPs).

### Participant recruitment

Participants were recruited using a purposive sampling method from two tertiary care Canadian pediatric hospitals between April 2021 and January 2022. The Hospital for Sick Children (Toronto, Ontario) and Stollery Children’s Hospital (Edmonton, Alberta) saw 80,000 and 60,000 patients, respectively in 2021. Neither of these institutions uses SARs in their ED, however, the Nao robot (without AI enhancement) has been used for research purposes at both institutions. Eligible children included 5–11-year-olds who presented to the pediatric ED and received an IVI. This age group was selected because they report higher levels of pain and distress during invasive procedures, can reliably self-report pain, and, developmentally, are most likely to benefit from immersive distraction tools [25–27]. Eligible caregivers were individuals accompanying a child who met the inclusion criteria above and were able to be interviewed in English. Eligible participants were identified through the ED electronic patient tracking system and approached by a research assistant. Those interested underwent informed consent after the IVI was completed. All study records were stored using Research Electronic Data Capture (REDCap) [28], a secure online data collection platform. All participants signed an online consent form; children unable to consent provided assent.

### Data collection

Caregivers (for themselves and on behalf of their child) completed an online questionnaire via REDCap collecting information on demographics and previous technological experience prior to the start of the interview or focus group. Interviews, either individual or focus group, were conducted virtually via Zoom Healthcare by two female team members (SH, FN); SH is a medical student and FN is a clinical research project coordinator, both are trained in qualitative interviewing and were supervised by senior team member (JS) with expertise in qualitative research and co-design. Interviewers did not have a clinical or personal relationship with any participants. Interview questions were developed based on the team’s expertise in conducting previous needs assessments and included questions to comprehend (1) children’s and caregivers’ experience during IVI, (2) thoughts about AI technology, including ethics, and (3) ideal features and functionality of an AI-enhanced robot (Supplementary Document 1 for interview guide). Prompts were incorporated as needed to clarify and encourage expansion of responses.

### Qualitative analysis

Audio interview recordings were transcribed verbatim. Following a content analysis approach [24,29], a subset of transcripts was reviewed by three team members (FN, AK, PP) with the aim of creating a coding scheme. Following this, all data was entered into Dedoose (Version 9.0.46), to facilitate coding. Two team members (AK, PP) independently coded an initial set of five transcripts after which themes were discussed leading to the development of supplementary codes to adequately capture all data. The remaining transcripts were independently coded by two individual team members (AK, PP). Following completion of coding, quotation review was conducted to extrapolate content areas, with input from all team members.

## Results

Fifty-nine families were screened or approached for consent at Stollery Children’s Hospital, and at the Hospital for Sick Children, 49 families were screened or approached for consent. Nineteen children and 11 caregivers were recruited from both sites. Three children from the 8–11-year-old age group participated in a focus group interview, while the remaining child participants and all caregiver participants were interviewed individually. Most children were female with a mean age of 8.4 (SD 2.2) years. All caregivers (11/11) were mothers, with 7/11 (63.6%) aged 40-49 years (See Tables 1 and 2). Only one child and one caregiver had previous experience with a SAR in medical setting.

**Table 1.** Demographic, health characteristics, and technological experience of children

Characteristics	Children (*n* = 19)
Age in years, mean (SD)	8.4 (2.2)
Sex, *n* (%)	
Female	14 (74)
Male	5 (26)
Race, *n* (%)	
White	11 (58)
Mixed Race	3 (16)
Other*	5 (26)
Reason for hospital visit, *n* (%)	
Gastrointestinal	3 (16)
Musculoskeletal	3 (16)
Infectious	3 (16)
Hematologic	3 (16)
Respiratory	2 (10)
Metabolic	2 (10)
Neurological	2 (10)
Multiple reasons	1 (5)
Previous IV, *n* (%)	16 (84)
Experience with voice assistant (e.g., Siri), *n* (%)	16 (84)
Experience with home assistant (e.g., Google Home), *n* (%)	14 (74)
Experience with robot technology (e.g., Roomba), *n* (%)	7 (37)

* Other includes: Indigenous (n = 1), Arab/West Asian (n = 1), Black (n = 1), Chinese (n = 1), South Asian (n = 1).

**Table 2.** Demographic, health characteristics, and technological experience of caregivers

Characteristics	Caregivers (*n* = 11)
Caregiver/Child relationship, *n* (%)	
Mother	11 (100)
Sex, *n* (%)	
Female	10 (91)
Male	1 (9)
Age (in categories), *n* (%)	
30–39 years old	4 (36)
40–49 years old	7 (64)
Race, *n* (%)	
Indigenous (Inuit, First Nations, Métis)	1 (9)
Black	1 (9)
White	8 (73)
Mixed Race	1 (9)
Income, *n* (%)	
>$90,000	6 (55)
Prefer not to answer	5 (45)
Highest level of education, *n* (%)	
High School	1 (9)
College/University	8 (73)
Professional/Graduate Degree	2 (18)
Experience with voice assistant (e.g., Siri), *n* (%)	4 (36)
Experience with home assistants (e.g., Google Home), *n* (%)	6 (55)
Experience with robot technology (e.g., Roomba), *n* (%)	6 (55)

The insights collected from children and caregivers were categorized into three major themes: (1) experience in the clinical setting, (2) acceptance and concerns surrounding SAR, and (3) SAR features to support child engagement (See Table 3).

**Table 3.** Description of themes and subthemes with supplemental quotations for child and caregiver interviews in the emergency department (ED)

Theme, subtheme, and description	Example of statements
**1. Experience in the clinical setting**	
1.2 Child and caregiver anxiety	
Child anxiety in the ED	“*What made [it] worse was whenever I had to think about going to the hospital, I would get anxiety, because I would probably have to get needles”* - [Participant 10, 11 years old]
Previous negative experiences with IVIs	*“My daughter is deathly scared of IVs, because she’s had quite a few in the last year and a half. So, for her, it’s not a great experience. I think for all of us in general, it’s not a great experience. Obviously it hurts, [but] for her she’s just completely traumatized by it, so it’s not a fun situation to deal with”* [Participant 6, Caregiver].
Caregiver perspective of managing child distress and anxiety in the ED	“*Not only is she undergoing stress, I’m under a huge stress as a parent, and then, I was worried that she was going to kick them [clinical staff] or hit them [clinical staff].*” - [Participant 6, Caregiver]
1.3 Pain and distress management tools	
Tools to manage pain and distress in the ED	“*We watched Paw Patrol on Netflix…we brought books from home.*” - [Participant 11, 5 years old]
Limitation to technological tools in the ED	*“Better Wi-Fi100% would have made it [ED experience/IVI procedure] better.”* [Participant 25, Caregiver]
Caregiver advocacy in the ED to effectively manage their child’s pain	“*I said, can you please just do “up the nose” [i.e., sedation] so we don't go through what we went through the last time, and he [HCP] goes, “absolutely.” He [HCP] could see the stress on my face, and on my daughter’s face, and I think that added a little bit of relief.*” - [Participant 6, Caregiver]
Caregiver perspective of providing distraction and distress management can	“*It was hard for me to do both distraction and comfort*.” - [Participant 12, Caregiver]
Positive presence of HCP	“*We got the freezing spray. That definitely helped and she said that it felt more like pressure when they were doing it, at least for the first needle she got.*” - [Participant 7, Caregiver]
**2. Acceptance of and concern surrounding SARs**	
2.1 Knowledge and acceptance of SARs	
General perception of robots	*“They’re machines that help people.*” - [Participant 15, 7 years old].
Caregiver vision of the SAR	*“I think it probably would help the parent too because you’re then interested in your kid’s interaction with the robot, so it might distract and bring down everybody’s anxiety”* - [Participant 7, Caregiver] *“If they could do tricks or they told jokes or they sang or had music options or dancing options”*- [Participant 2, Caregiver] *“A lot of kids, they’re very innocent, and technology is going to be used in an innocent way… [they’ll be] a lot more trusting.”* - [Participant 23, Caregiver]
Child vision of the SAR	*“Maybe dance or sing or just, try and talk about a different topic with you.”*- [Participant 21, 11 years old]
2.2 Concerns with robots	
Caregiver concern about photographing/video recording by the SAR.	*“…If it was a trauma or burns or something far more severe, then I wouldn't be comfortable with that being recorded.”* - [Participant 7, Caregiver] *“Not having a video camera watching my child kind of thing.”* - [Participant 12, Caregiver]
Child concerns about technical failure of SAR	*“I wouldn't really think so, unless something really gone wrong and it started knocking things down by accident or something like that”*- [Participant 21, 11 years old].
**3. SAR features to support child engagement**	
3.1 Role of SAR	
New roles for the SAR outside the IVI	*“With wait time, like it could be distracting, because the hardest part is waiting.”* -[Participant 7, Caregiver].
Caregiver perceptions of role of the SAR	*“Maybe it could ask the child or have a repertoire of things that it can do and give the child maybe two or three choices to pick from”* - [Participant 17, Caregiver].
Children perceptions of role of the SAR	*“Pretend that it was getting the needle too so then it would be like we’re getting it together”* - [Participant 6, 8 years old].
3.2 Robot features	
Friendly features	*“He was very friendly, kind…because I see the face and the mouth…and giving them a high five and saying hello and helping them…”* - [Participant 16, 6 years old]
Scary features	*"A nice robot would just be a plain robot that didn't have any tools that are scary"* - [Participant 29, 11 years old]
Children recognize that the SAR was programed	*“Lots of robots are built to do certain things like mechanical stuff, which makes robots smart. Also you could program it have a brain or something”* - [Participant 21, 11 years old]
Intelligence features	*“It could tell that the girl was scared and didn't want to get a needle.”* - [Participant 27, 8 years old]
3.3 Feature enhancement	
Caregivers suggested enhancement	“*If the child was crying or upset, I wouldn't want the robot to necessarily cry and be upset, that might be more upsetting, but being able to then display comfort or empathy.*” - [Participant 7, Caregiver]

HCP = healthcare provider, SAR = socially assistive robot.

## Experience in the clinical setting

### General experience in ED

“*For me I would love to have an iPad or something available for the kids, for every room. Obviously, it’s probably not feasible, but something like that to help distract them if they need it.*” – [Participant 25, Caregiver]

While most children and caregivers were comfortable in the ED environment, some common elements of discomfort were identified. These included long wait times, sensory discomfort associated with bright lights, cold rooms or uncomfortable chairs and beds, and a lack of technological support (e.g., iPads, TVs, chargers).

### Child & caregiver anxiety

“*I was scared when I was in the emergency room because it had a poster and it said, “Is your child getting a needle” and then I told my mom, “Am I getting a needle, mom?” and then she said, “Probably.” I had butterflies in my tummy, I was nervous*” - [Participant 28, 9 years old]

Nearly all children mentioned a fear or dislike of needles. Both children and caregivers emphasized the importance of addressing child anxiety and fear associated with needles, regardless of the additional time this may take. For some, these anxious feelings begin before they arrive at the ED.

Children shared experiences of how HCPs helped reduce their procedure-related anxiety and played an important role in their ED experience.

“*I felt kind of scared, because I didn't know if it was going to hurt or not. And then she [nurse] explained how all of it would work, and after that, it got more comfortable.*” - [Participant 20, 11 years old]

Children also shared how previous negative experiences with IVIs can impact their perception of future IVI encounters. This was also echoed by caregivers who described their children as “traumatized” by IVI.

“*I had this one really bad experience, and then I'm scarred for life, so I don't like needles.*” - [Participant 10, 11 years old]

Caregivers also reported that managing their child’s distress and anxiety can be a stressful experience for them.

### Pain & distress management tools

“*I felt really nervous but most of them, doctors or nurses, were very nice and they comforted me, and they helped me out. Then I felt really okay.*” - [Participant 13, 11 years old]

Common tools used to manage pain and distress include pharmacological tools (e.g., topical anesthetics), non-pharmacological tools (e.g., electronic devices, toys), and caregiver support techniques (e.g., comfort positions). Digital tools (e.g., iPads, smartphones) were most cited as helpful; caregivers reported using these to play games, watch shows (e.g., Netflix, YouTube), or listen to music – all with the aim of distraction. Children and caregivers also described non-digital tools (e.g., books, stuffed animals, blankets, coloring books) as providing comfort. One limitation of digital tools was the lack of or often unreliable internet access and unavailability of charging stations.

Several children named their caregivers as important in their pain and distress management.

“*Whenever I look at Mama or talk to her, it just feels [like] a little poke. But when I don't talk to Mama or look at her, then it hurts.*” - [Participant 15, 7 years old]

Caregivers also reported advocating for their children, as they often know what tools are most effective to manage their child’s pain and distress. Notably, some caregivers felt it was difficult to meet the multiple needs of their child during the procedure. Caregivers appreciated HCPs knowledge of pain management options. Their (HCP) presence and behavior when positively perceived by children can also help manage pain and distress.

## Acceptance of and concern surrounding SARs

### Knowledge and acceptance of SARs

“*If I was doing bloodwork and the robot was there with me, I would feel calm and relaxed and wouldn't be worried about it.”* - [Participant 24, 7 years old]

Children interviewed generally perceived robots as helpful. They had a good understanding of the general appearance of an archetypal robot, “made of metal,” “square head, square body.” Children viewed the SAR as a trusted tool and explained that they would communicate with it as they would communicate with their caregivers. Children and caregivers expressed interest and excitement about interacting with a SAR in the pediatric ED. Caregivers envisioned the SAR as a tool to distract their children and reduce anxiety during the IVI. Caregivers understood that children would likely perceive the SAR positively because of their young age and innocence. Among caregivers, the sense of trust was noted to be with the institution and/or people involved in developing the SAR rather than the instrument itself.

“*I would probably be very cautious but I’d also assume, because I trust the doctors and nurses that I’m putting the care of my child into, [that] they wouldn't be bringing something in to the ER that would be harmful – So it would be more the trust of the people that are using these tools and these robots.*” - [Participant 17, Caregiver]

### Concerns with robots

“*With somebody’s cry, it might sound more like laughter, or something like that. Somebody’s emotional face could be misinterpreted. Also, some people – I’m just going off of personal experience – even when I’m hurting really bad, I will laugh because I feel uncomfortable.*” - [Participant 23, Caregiver]

While all participants largely supported the idea of having a SAR in the ED, caregivers expressed some concerns associated with the adaptability of the SAR as well as privacy concerns related to photographing/video recording by the SAR. Caregivers thought the SAR might have difficulty interpreting a child’s social cues or facial expressions appropriately and the nuance required to understand a complex social setting, such as the ED. Caregivers also highlighted that some children, particularly younger children, may be overwhelmed by a SAR and it may therefore need to adapt its behaviors for each child.

Some caregivers expressed concern about sharing personal information and protecting their children’s privacy, in the context of SARs. They highlighted potentially inappropriate times for video recording (e.g., severe injury, seizures). Caregivers also emphasized the importance of having a consent process with proper explanation to them and their children prior to SAR use. Some caregivers did not want the interaction with the SAR and their child to be recorded at all.

“*Obviously, things are recorded for perfecting purposes, but that for me is something that freaks me out…but I know that it is for beneficial purposes. Maybe a confidentiality agreement. Signing a form stating that it’s [video footage] not going to be published anywhere, only for educational purposes, could benefit people in the future.*” - [Participant 23, Caregiver]

Children shared concerns about the technical failure of the SAR and its implications, such as losing control. There was one child who preferred interacting with nurses and doctors instead of interacting with robots.

*“I would prefer nurses. This is not anything bad about the robot. I just feel like it’s better…Some kids are scared of them and they do not know how to react to them. Maybe you want human beings instead of robots….I would say I trust more in humans than robots because robots are not going to be like, “I”m sorry.’ They aren't going to comfort you”* [Participant 13, 11 years old].

## SAR features to support child engagement

Children and caregivers perceived the role of the SAR differently; caregivers emphasized the role of the SAR during IVI, while children emphasized its importance before and after IVI.

### Role of SAR

“*It depends on the age of the child. For my daughter, I think her age group would be okay for it to just come in at the same time as the procedure is being done. But for a younger [child], I think she would need to get comfortable with it first.*” - [Participant 7, Caregiver]

Neither children nor caregivers suggested a set amount of time for the SAR to interact with patients, rather they suggested it stay if it was beneficial. Some caregivers stated that younger children might require more time with the SAR to get comfortable prior to IVI beginning. New roles were suggested for the SAR outside of IVI entirely, including having a SAR in the waiting room to distract children.

#### Caregiver perceptions of role of the SAR

“*If he had said to the robot, “what’s going to happen to me?,’ the robot probably would have [said], “this is exactly what’s going to happen step by step.’ Give him a bit of a heads-up. Maybe the robot and the kid made a plan together about what they’re going to do during the IV procedure.*” - [Participant 25, Caregiver]

Caregivers saw the primary function of the SAR as distraction during IVI. Suggested behaviors during IVI included tricks, jokes, playing music, singing, and dancing. It was suggested that SARs have a list of activities to pick from to better engage with children. Caregivers suggested that conversations with the SAR before IVI center around explaining the procedure and creating a distraction plan with the child about what should occur during IVI. Conversations after the IVI were suggested to cover what to expect next.

#### Children’s perceptions of role of the SAR

“*I think we should be talking. He can be helping me out [with]what I'm gonna be doing today. Like if I'm going for an ultrasound, what’s gonna be happening with me? What’s going on? I'm gonna have to stay there. To tell me what’s gonna happen and he'll be there for support.*” - [Participant 8, 8 years old]

From the child’s perspective, the SAR was viewed as a friend that would complete the procedure with them. Children emphasized the role of the SAR before and after IVI. Before, children wanted the SAR to talk to them about their procedure, get to know them, and play games. After the IVI, children thought the SAR should stay in the room and continue talking to them, while also providing positive reinforcement. Examples of supportive phrases include: “*you did such a good job or like you did really well*” (Participant 21, 11-year-old, SickKids), and “*you’re very brave*” (Participant 27, 8-year-old, Stollery). Children also suggested being rewarded after IVI; the SAR could give them a prize, gift, or sticker. The before and after periods may be important to children because some notice that when the HCP enters the room, the focus can shift to the medical procedure and the SAR may then be limited in the actions it can take.

“*The robot wanted to come and play with me. But when the doctor’s coming he [the SAR] can't play with me, “cause if the doctors give me the IV and he [the SAR] wants to walk, I can't because I’m hooked up to the IV…the doctor said that I have to stay in bed.*” - [Participant 16, 6 years old]

### Robot features

All participants were played a 50 s video of a Nao robot (without AI-enhancement) helping a child through a needle procedure (video can be seen here). Participant responses to the video outlined their opinions on which features would make the SAR friendly, funny, smart, or scary.

#### Friendly features

“*This voice is calm and soothing. It was moving. It wasn't making noise, it wasn't beeping. Smooth lines and the colours. It was talking to her like it knew what she was doing at the time and was talking her through it. [It] introduced itself and used her name.*” - [Participant 12, Caregiver]

To characterize a friendly SAR, the suggested features included both physical design and behaviors. Children listed having a “nice” voice, smiling, and bright colors for the external appearance of the SAR. Behaviorally, they suggested introducing oneself, knowing the child’s first name, offering help, and giving high-fives.

#### Comical features

“*Dressing [in] funny clothing or make it wear a hat?*” - [Participant 21, 11 years old]

Participants explained that telling jokes, having a funny name, acting silly, dancing, and having funny voices are features that would make a robot funny. Children also mentioned costumes as another method to make the SAR appear funny.

#### Scary features

When asked about what makes a robot scary, participants identified having red eyes, being large in size, dark in color, having a deep voice, and sudden movements. One child perceived tools as scary.

#### Intelligence features

“*He knew what it [IVI] was like – it’s wet for a bit, it’s gonna feel tight for just a second, but then as soon she takes it off, it’s gonna go away.’ He felt smart, like he knew what was gonna happen.*” - [Participant 8, 8 years old]

Most children thought the SAR was smart and some recognized that the SAR was programed with designed behaviors. Participants thought a smart SAR should be knowledgeable about a variety of topics and be able to converse with children about these, appropriately recognize and respond to needs of the child, and know the IVI procedure well to explain and respond to a child about it.

### Feature enhancement

*“It didn't really show a lot of feelings. Just kind of a straight voice… it would be fun if they could change his voice, [like] if he was excited”* [Participant 29, 11 years old].

Participants identified areas where the SAR features could be enhanced to meet the needs of children undergoing IVI procedure, either physically or behaviorally. Several children suggested feature enhancement to the color of the robot, including potential for different colors or letting a child pick a color of their choice. Voice enhancements were also suggested, including clarity, and making the SAR’s voice more animated. Participants also preferred the SAR to stand instead of sit and suggested adding more movement to capture a child’s attention.

*"Definitely movement, in order to keep a kid’s attention, I think it needs to be moving and making noises"* - [Participant 2, Caregiver].

Caregivers suggested tailoring language and distraction activities to match the child’s developmental level to maximize effectiveness.

*"What’s age appropriate for a four-year-old is not going to be a good distractor for a 12-year-old. Is it able to say something helpful age appropriate for that child and their interests?"* - [Participant 7, Caregiver].

They also recommended the robot have the ability to adapt to the child’s needs and communicate these, as they may not be perceived during the IVI.

## Discussion

This study highlights the perspectives of children and caregivers surrounding the use of AI-enhanced SARs in pediatric healthcare. Participants shared insights on their previous experiences in pediatric ED settings, as well as their thoughts on the use of AI-enhanced SARs within these spaces, specifically indicating perceived benefits, concerns, and desired features. Several implications were identified for the design and development of AI-enhanced SARs within pediatric healthcare settings, which are applicable to this project as well as other similar technologies in related applications.

### Comparison to previous literature

Two previous studies completed co-design with children to inform the design and development of their SAR [21,23]. Overall, both studies highlight the value of the co-design process. Similar to our findings, these studies reported that movement or dynamic behaviour was integral in the SAR’s ability to be engaging [21,23]. In terms of the SAR behavior, being knowledgeable, friendly and helpful were important, for the physical appearance of an SAR the color or ability to change color was important to children [21,23]. However, none of these studies were implemented in a clinical setting, therefore some of their results were not relevant to the ED context. To our knowledge, no studies have described co-design involving caregivers to inform the design and development of their SAR.

### Clinical implications

The experiences and perspectives shared by participants reinforce the findings of published evidence which indicate the importance of supporting children through IVI [30–32]. Children and caregivers identified unique roles for a SAR throughout the phases of IVI. Across the interviews, caregivers emphasized SAR utility before and during IVI, when anxiety levels are highest and have greatest potential to disrupt procedure flow and bolster formation of negative procedure-related memories [33]. Prior to IVI, both children and caregivers expressed a desire for the SAR to explain the upcoming procedure, thereby allowing time to create and solidify a shared distraction plan prior to healthcare providers entering the room, which often causes anxiety to sharply rise. Previous work indicates the importance of pre-procedure communication about IVI [30]. In this study, most caregivers reported feeling well-informed about the procedure after discussion with their HCP but expressed a wish for their child to be treated as someone who will understand and receive adequate explanation of the procedure and what to expect. We propose that by augmenting this task with a SAR, there is potential for its inherent novelty, associated excitement, and array of distraction options to enhance children’s understanding and reduce pre-procedure anxiety.

During IVI, caregivers and children emphasized the importance of providing a wide variety of distraction options and anticipated positive value in providing a repertoire of choice given how children of varying ages, backgrounds, and personalities likely prefer different distraction techniques [34]. Furthermore, as with any medical intervention, caregivers mentioned the importance of ability to easily pivot among a wide array of options may be beneficial during IVI if a chosen option proves ineffective in reducing distress. Evidence suggests effective distractions focus on choice, and the adaptability of the proposed SAR will allow for seamless pivoting to empower children with options [27].

Following IVI, children expressed a wish for positive reinforcement by the SAR. Post-procedure de-briefing and re-framing is supported by a wealth of evidence as critical in positively framing a child’s immediate perception and long-term memory of procedure-related pain and distress [35]. After IVI, the proposed system would likely be able to remain in the patient room longer than HCPs and could adaptively undertake real-time debriefing conversations with children and families to facilitate positive re-framing and memory formation, thereby lessening long-term procedure-related anxiety. While not the objective of the currently presented study, the indication of SAR utility within this unpredictable setting highlights an important opportunity for expansion of inherently adaptable AI-enhanced SAR applications within this healthcare space. Waiting room applications have potential to further bolster children’s positive perceptions of the healthcare experience.

### Technical & ethical implications

Previous research evaluating the implementation of social robots in healthcare settings found personalization as an important enabler of use [36]. Children and caregivers in our study spoke of similar needs. Personalization includes tailoring behavior to meet the needs of each child while also being able to adapt to the unique healthcare context. Furthermore, children and caregivers suggested the SAR should be able to interpret a variety of social cues and facial expressions. The need for age-specific tailoring is important for younger children who may visibly display distress rather than vocalizing it. This need also underscores the ethical dimension of this project; literature on human-robot interaction indicates that equity is an important aspect of SAR design [37,38]. Machine-based prioritization of certain languages, accents, or even skin color, has been a major problem in some previous AI-generated tools, and cannot be tolerated [39–41]. All machine-learning components of this system will be trained on data from the target deployment locations, which are both large urban EDs with diverse populations, thereby ensuring that the final system functions well within the diverse realm of possible users.

Although most caregivers understood the reasoning for the SAR to undertake photographing/video recording, many expressed concern for their child’s privacy. As such, the implementation of the SAR in a pediatric ED setting must include a thorough explanation of how and why photo/video recordings are being collected and what privacy precautions will be in place. Collected visual and auditory input data will help the system understand its environment and interact accordingly but will never be stored; this will be made clear to all participants during the consent process. Conversely, certain carry-over effects were endorsed by caregivers who indicated that if they trusted the healthcare team, they would be more likely to trust the SAR. This underscores the importance of co-design in the creation and implementation of new technologies within healthcare settings. Other barriers exist in the implementation of a SAR, these include financial constraints, flexibility of SAR in a resource-strained environment, and uptake by HCP. These challenges will be thoroughly examined in a future publication, which examines the viewpoints of healthcare providers.

Finally, child participants implicated technical failure as a concern with using SARs. Technical barriers, such as system lag, interruption, or failure, were also identified as limitations to the implementation of the SARs in healthcare settings [36,42]. To mediate this, system robustness will be maintained by including a control panel that the operator can use to supplement the sensor information if needed. Furthermore, users will be provided with an explanation regarding the fallibility of machines. The SAR will be equipped with failsafe behavior to allow the operator to interrupt the robot’s behavior in the event that it’s understanding of the situation and resultant actions are incorrect and/or inappropriate.

### Limitations

Virtual interviews may have limited the ability of the interviewer to detect and interpret subtle body language and may potentially impede interactions between focus group participants. The sample is exclusively comprised of English-speaking families that presented to large Canadian urban EDs; as such, their perspectives may differ from those of families who were not comfortable attending in-person clinical care during the COVID-19 pandemic and/or spoke other languages, thereby potentially limiting generalizability. Finally, the study findings describe the design recommendations and perceptions of some end-users, these may not align with actual behaviors and responses of a SAR implemented in a real-world scenario.

### Future work

An initial prototype of the SAR system is currently being developed, with the system’s behaviors based on outputs of this co-design work. A parallel co-design study was undertaken to characterize the perspectives of healthcare practitioners, which is also being used to inform system design [43]. Following the initial prototyping stage, the iterative design process will continue with usability testing. When a viable SAR is complete, a two-site randomized controlled trial will be conducted to evaluate the efficacy of this system in decreasing pediatric pain and distress during IVI in the pediatric ED. Throughout the project, the social and ethical implications of using AI-enhanced SARs within pediatric healthcare are being explored by our multi-disciplinary team of ethicists, healthcare providers, engineers, computer scientists, computational linguists, and Indigenous language experts. These insights will guide how the role and limitations of SARs will be communicated to children and caregivers as well as inform future adaptations of the SAR to other languages and cultural contexts.

## Conclusion

Overall, AI-enhanced SARs were perceived by children and caregivers as a promising tool to distract children. Insights collected will be used to inform the ethical and emotionally safe design of an AI-enhanced SAR. Next steps include development and usability testing of the SAR, subsequent evaluation in the pediatric ED via a randomized controlled trial, and clinical implementation. Those wishing to characterize the needs of children and caregivers within similar healthcare scenarios are invited to apply a similar approach.

## Supporting information

Nishat et al. supplementary material Nishat et al. supplementary material

## Supplementary material

The supplementary material for this article can be found at https://doi.org/10.1017/cts.2023.608.
