# Supplementary material for: Exploring the needs of children and caregivers to inform design of an artificial intelligence-enhanced social robot in the pediatric emergency department
Source: J Clin Transl Sci. 2023 Aug 24;7(1):e191. doi: 10.1017/cts.2023.608 (PMC10514688; doi:10.1017/cts.2023.608)
Supplement: Nishat et al. supplementary material [file S2059866123006088sup001.pdf]

Semi Structured Interview guide (Children)

We are interested in learning more about how you feel a robot with artificial intelligence technology could be used to help kids in the emergency department. We are going to start off with some general questions about your experience in the emergency department, and then move into some more specific questions about how technology could help to support you or other kids going through a similar procedure. Please note that this interview/focus group will be audio recorded.

If focus group:
This focus group will last for about 45 – 60 minutes. The aim of the focus group is to get everyone's input on each question. We will begin with some general questions and give everybody a chance to speak. We value everyone's opinion and would appreciate if there are no interruptions while others are speaking. We are going to keep the conversation on track by giving each person a turn. If we hit a spot where someone has a lot to say or wants to add an additional point, we will put their name on this board (our "parking lot" of ideas) and at the end, I will come back to these issues.

If individual interview:
This interview will last 45-60 minutes. The aim of this interview is to get your input on each question. We will begin with some general questions and give you a chance to speak.

Broad Questions:

Think about the last time you were at the emergency department.

1. Can you tell me how you felt when you had to go to the emergency department (or hospital if child is young)?
   - What about when you had to get a needle?

2. What helps make the emergency department visit better, if anything?
   - Your parents? The Nurses or doctors? Any other staff? Any toys or games?

3. [Question for older kids]: Did you have any useful supplies that helped while in the emergency department?
   - What services would you like to have available to help you manage (cope, treat) your pain while in the emergency department?
     - Healthcare professionals, education, distractions, strategies to manage pain, etc?
   - Did you use anything on your phone to help? Music, games, etc?

4. What made the visit to the emergency department worse, if anything?

- Something a parent or doctor/nurse said or did? Anything that was confusing or scary?
- [For older kids: anything that was not explained or a procedure you were not told about, etc.]

5. Do you feel that there’s anything that could have made the emergency department visit better?
   - Anything at all – even if it seems far-fetched!

Next we’re going to talk about technology, specifically robots.

Warmth
1. How do you know if a robot is friendly?
2. Tell me about a funny robot/What makes a robot funny?
3. Do you think robots can have feelings?

Competence
4. How can you tell if a robot is smart?
5. How do you know if a robot sees you?
6. How do you know if a robot understands you?
7. Are there special things you’d only tell a robot? (and not a doctor or nurse)
8. Should the robot explain what it is doing?

Discomfort
9. How do you know if you can trust a robot?
10. What should happen if a robot misbehaves?
11. What makes a robot scary, sad, …?

[Show a video of Medi robot]

Broad
1. What did you like about the robot in the video?
   - Singing? Dancing? Talking? Walking?

Warmth
1. Was this a friendly robot?
   a. Why or why not?
   b. What could make this robot more friendly?
2. Was this robot funny?

a. Why or why not?
b. What could this robot do to be funny?

3. Do you think this robot has feelings?
    a. Why or why not?

Discomfort/Trust

1. Can you trust this robot? Why or why not?
2. Did this robot make you feel scared, or sad, or any other feelings?
    a. Why?
3. Are there any bad things about using a robot in the emergency department?

Competence

4. Was this robot smart?
5. Do you think this robot can see things?
6. Did this robot explain what it was doing?
7. What should a robot do to help while you're getting a needle?
    a. What would it say?
    b. What would it do?
    c. How could it help you feel better?

Other

1. Is there anything else you'd like to share with us about how a robot could be used in the emergency department?

Semi Structured Interview guide (Parents)

We are interested in learning more about how you feel a robot with artificial intelligence technology could be used to help kids during their visit to the emergency department. We are going to start off with some general questions about your experience with your child in the emergency department, and then move into some more specific questions about how technology could help to support your child or other children going through a similar procedure. Please note that this interview/focus group will be audio recorded.

If focus group:
This focus group will last for about 45 – 60 minutes. The aim of the focus group is to get everyone's input on each question. We will begin with some general questions and give everybody a chance to speak. We value everyone's opinion and would appreciate if there are no interruptions while others are speaking. We are going to keep the conversation on track by

giving each person a turn. If we hit a spot where someone has a lot to say or wants to add an additional point, we will put their name on this board (our “parking lot” of ideas) and at the end, I will come back to these issues.

If individual interview:
This interview will last 45-60 minutes. The aim of this interview is to get your input on each question. We will begin with some general questions and give you a chance to speak.

Broad Questions:

1. Can you tell me about your child’s experience having the IV (needle) procedure done?
   a. Was it painful for your child? What were the biggest challenges?

2. What helped make the emergency department visit better, if anything?
   a. Your presence, the nurses or doctors? Any other staff? Any toys or other distractions?

3. Did your child have any tools or resources that helped with the pain while they were in the emergency department?

4. Was a child life specialist ever involved during your visit?

5. What services would you like to have available to help your child manage (cope, treat) their pain while in the emergency department?
   a. Healthcare professionals, education, distractions, strategies to manage pain, etc.?
   b. Did your child use anything on their phone to help? Music, games, etc?
      i. What is the most distracting game/toy/phone app for your child and why?

6. What made the visit to the emergency department worse, if anything?

7. Something anyone said or did? Anything that was not explained or given notice, etc.

8. Do you feel that there’s anything that could have made the emergency visit better?
   a. Anything at all – even if it seems far-fetched!

9. Do you think managing your child’s pain during an emergency department visit, with or without a robot, is an important priority?

10. Do you think managing your child’s pain during an emergency department visit, with or without a robot, delays care or extends the length of stay during emergency department visits?

Next we're going to talk about technology, specifically robots and artificial intelligence. Artificial intelligence is the science and engineering of making machines that can perform computational tasks which normally require human intelligence. This can include a humanoid robot for procedural distraction in emergency department or other settings that you have interacted with.

For the following questions, lets imagine a robot in the room with you during your visit to the emergency department.

Warmth

1. Do you think your child could see a robot as being friendly?
2. How could a robot show that it is friendly?

Competence

3. How can you tell if a robot understands what is going on during your visit to the emergency department?
4. What should a robot do to distract your child?
    a. How could this be helpful during your child's IV procedure, if at all?
    b. What would the robot do/say/how would it move to show that is being helpful during the IV procedure?
5. Should the robot explain what it is doing?

Discomfort

6. What concerns might you have about your child interacting with robots?
7. How do you feel about a robot that will be providing (emotional) support? Why do you feel that way?
8. How do you know if you can trust a robot?
9. Would you be comfortable with a robot copying a child's expressions and emotions?
10. Is it okay for the robot to use information from interactions with your child to train itself to better respond in future situations?
    a. For example, the robot recognized that your child was happy when it sang a song. It would then train itself and play a song for children who are sad in future situations.

Other

11. When trying to distract your child from something unpleasant/painful what methods do you normally use?

12. Do you have any other thoughts about how a robot could be used in the emergency department before we show you a video of an example of a robot?

[Show a video of Medi robot]

Now that you've seen an example of one type of robot, we'll ask you some questions about robots in children's healthcare.

Warmth

2. Do you think this robot is seen as being friendly?
    a. Why or why not?

Competence

3. Do you think this robot would be helpful to distract your child while in the emergency department?
    a. Why or why not?
    b. What about this robot do you see as being helpful? E.g., what the robot does/says, or how it moves?
4. Is there anything else a robot could do in the emergency department to be helpful for your child?

Discomfort

5. Is there anything you don't like about this robot?
    a. How it looks/ how it moves/ how it makes you feel?
6. Now that you've seen an example of a robot, do you have any other concerns about having your child interacting with robots?
7. Can you imagine this robot as being able to provide (emotional) support?
    a. Why or why not?

Other

8. Is there anything else you'd like to share with us about how a robot with artificial intelligence could be used in the emergency department?
